# Association between Dietary Intake and Faecal Microbiota in Children with Cystic Fibrosis

**DOI:** 10.3390/nu15245013

**Published:** 2023-12-05

**Authors:** Jazmín Viteri-Echeverría, Joaquim Calvo-Lerma, Miguel Ferriz-Jordán, María Garriga, Jorge García-Hernández, Ana Heredia, Carmen Ribes-Koninckx, Ana Andrés, Andrea Asensio-Grau

**Affiliations:** 1 University Institute of Food Engineering (FoodUPV), Polytechnic University of Valencia, Camino de Vera s/n, 46022 València, Spain; 2 Joint Research Unit NutriCuraPDig, Avda. Fernando Abril Martorell 106, 46026 València, Spain; 3 Cystic Fibrosis Unit, University Hospital Ramón y Cajal, M-607, 9, 100, 28034 Madrid, Spain; 4 Advanced Food Microbiology Centre (CAMA), University of Valencia, Camino de Vera s/n, 46022 València, Spain; 5 Health Research Institute La Fe, Celiac Disease and Digestive Immunopathology Unit, Hospital Universitari i Politècnic La Fe, 46026 Valencia, Spain

**Keywords:** diet, cystic fibrosis, microbiota, dietary fibre, fat

## Abstract

A “high-fat, high-energy diet” is commonly recommended for children with cystic fibrosis (CF), leading to negative consequences on dietary patterns that could contribute to altered colonic microbiota. The aim of this study was to assess dietary intake and to identify possible associations with the composition of faecal microbiota in a cohort of children with CF. A cross-sectional observational study was conducted, including a 3-day food record simultaneously with the collection of faecal samples. The results showed a high fat intake (43.9% of total energy intake) and a mean dietary fibre intake of 10.6 g/day. The faecal microbiota was characterised at the phylum level as 54.5% Firmicutes and revealed an altered proportion between Proteobacteria (32%) and Bacteroidota (2.2%). Significant associations were found, including a negative association between protein, meat, and fish intake and Bifidobacterium, a positive association between lipids and *Escherichia/Shigella* and Streptococcus, a negative association between carbohydrates and *Veillonella* and *Klebsiella*, and a positive association between total dietary fibre and *Bacteroides* and *Roseburia*. The results reveal that a “high-fat, high-energy” diet does not satisfy dietary fibre intake from healthy food sources in children with CF. Further interventional studies are encouraged to explore the potential of shifting to a high-fibre or standard healthy diet to improve colonic microbiota.

## 1. Introduction

In patients with cystic fibrosis (CF), multiple gastrointestinal tract disorders and pancreatic insufficiency lead to maldigestion and malabsorption of nutrients as a consequence of the genetic defect in the CFTR protein [1]. The disease causes the accumulation of mucus, leading to obstruction of the pancreatic duct and preventing the secretion of digestive enzymes. So, the need arises for supplementation with pancreatic enzymes that allow the luminal digestion of nutrients, especially dietary fat [2]. To counteract energy losses and the disease pathogenesis itself, the classic “high-fat, high-energy” diet has been recommended [3]. However, concerns have been raised lately regarding this type of diet. In most cases, children with CF have unhealthy dietary habits as energy requirements are met with foods with high contents of saturated fat and simple carbohydrates [4]. Additionally, the scarce intake of fruit, vegetables, nuts, and legumes linked to this diet hinders reaching the target of dietary fibre intake for the general child population [5]. However, highly effective CFTR modulators are the main reason for reconsidering the continuation of the “high-fat, high-energy diet” as a mainstream element in treatment. Modulators are a new class of drugs that directly target molecular defects in the CFTR protein to increase CFTR activity and, in recent years, have been shown to lead to overall improved disease outcomes and prognosis [6]. In spite of the advantages, depending on the type of mutation and the type of modulation therapy used, the treatment can result in the accelerated development of overweight and obese in this population if the high intake of fat and energy is maintained [7]. 

Another well-defined disease complication is cystic fibrosis-related gut dysbiosis (CFRGD). Dysbiosis is characterised by a reduction in the diversity of microorganisms in the intestinal microbiota, an imbalance between beneficial and pathogenic bacteria [8], and the deficient production of metabolites that help regulate the organism, including short-chain fatty acids (SCFAs) [9]. Previous studies on faecal microbiota in CF concur in reporting a decreased Bacteroidota phylum along with increased Proteobacteria [10], including the overrepresentation of certain genera in the *Enterobacteriaceae* family [11,12,13]. Other studies highlighted the decrease or the absence of some potentially beneficial bacteria, including *Akkermansia*, *Faecalibacterium*, and *Roseburia* [8,14,15,16]. Currently, the scientific literature only reflects a few attempts to improve gut dysbiosis in CF, including the use of CFTR modulators (Ivacaftor), which are sown to increase the relative abundance of *Akkermansia* [17]. Other studies addressed the potential of probiotic supplementation, but few of them focused on the changes in colonic microbiota [18].

Overall, CFRGD could be, in part, related to the specific dietary habits of children with CF, as in other contexts of high-fat diets, and could be modulated through adequate dietary patterns [19]. However, there is no evidence of the impact of following an alternative diet to the conventional “high-energy, high-fat” dietary prescription in the context of CF.

Some components of the diet, such as dietary fibre, have not been duly considered in previous studies on the dietary habits of children with CF, when the fact is that dietary fibre has been shown to exert beneficial effects on colonic microbiota in the general population [20]. Thus, proposing healthier diets with a focus on dietary fibre could be an effective approach to revert CFRGD. However, it is first needed to generate evidence on the real impact of the “high-fat, high-energy” diet on colonic microbiota in terms of how specific dietary components and their food origin impact specific microbial groups and their metabolic activity. Establishing this background would help guide future interventional research studies on the diet of children with CF.

Therefore, this study aimed to explore possible correlations between diet, gut microbiota profile, and metabolites, such as the production of SCFA, and to identify dietary fibre types as potential modulators of CFRGD.

## 2. Materials and Methods

### 2.1. Subjects and Study Design

A prospective, cross-sectional, observational study was carried out on 43 paediatric patients with CF in follow-ups at the Paediatric CF Unit of the Hospital Universitari i Politècnic La Fe (Valencia, Spain) and Hospital Universitario Ramón y Cajal (Madrid, Spain) enrolled in the MyCyFAPP Project (H2020, 643806). The study protocol and patient information sheets together with the informed consent were approved by the respective ethics committees (Ref. 2021-111-1) in May 2021. The informed consent form was signed by one of the parents of each participant and also by the participants who were over the age of 12. For this sub-study, the participants were required to have a 3-day food record and a stool sample. The faecal samples were stored in freezers at −20 °C until analysis. The clinical data were collected by accessing the medical records of the patients, including age, sex, weight, height, mutation, pancreatic insufficiency, CFTR modulator therapy, and antibiotic use. To store the data, the eCRF platform specifically created for clinical research studies, MyFoodREC (https://remote.iislafe.san.gva.es/myfoodrec (accessed on 1 March 2023), was used.

### 2.2. Nutritional Data Collection and Processing

The 3-day food records included the time of intake, the name of the meal, and the amount of food and were filled in by the parents of the participants in the study. The data were entered into the MyFoodREC platform, which has the necessary tools to calculate the composition of the diet in energy and nutrients. The food intake was also expressed in terms of food groups. A total of 16 food groups were established according to the classification used in a previous study with slight modifications [21]: milk and dairy, sugar-added dairy, fruit, vegetables, legumes, nuts, whole-grain cereals, refined cereals, snacks and sweets, meat, cold meats, fish, eggs, oils, butter/margarine, and pastries (Table 1). Also, a specific database was developed to evaluate the consumption of different types of dietary fibre (hemicellulose, pectin, cellulose, and lignin) and its origin according to the food group; for heterogeneous foods, the calculation was made considering each ingredient. The data used for the calculation were taken from [22] (Table S1).

### 2.3. Analysis of the Microbiota and Its Metabolic Activity

#### 2.3.1. Microbiota Composition by 16S rRNA Amplicon Gene Sequencing

Total DNA was extracted from all the samples using the Stool DNA Isolation Kit from Norgen Biotek Corp^®^ (Thorold, ON, Canada), following the manufacturer’s protocol and recommendations. The final yield of the extracted DNA was determined by fluorometry (Qubit fluorometer, Invitrogen Co., Carlsbad, CA, USA). V3-V4 hypervariable regions of the bacterial 16S rRNA gene were amplified using aliquots of the isolated DNA from each sample. Amplicons were checked with a Bioanalyzer DNA 1000 chip, and libraries were sequenced using a 2 × 300 bp paired-end run (MiSeq Reagent kit v3) on a MiSeq-Illumina platform (FISABIO sequencing service, Valencia, Spain).

The sequences obtained by sequencing on the Illumina MiSeq platform (2 × 300 bp) were filtered for subsequent analysis. Filtering and quality assessment were performed in FISABIO sequencing service using the fastp program [23], based on quality (removal of low-quality nucleotides at the 3′ end by 10 nucleotide windows with an average quality score under 30) and length (removal of sequences with less than 50 pb). R1 and R2 from Illumina sequences were joined using FLASH program [24], applying default parameters. In order to analyse the bacterial community by amplicon sequence variants (ASVs), the joined data were processed in dada2 package (version 1.26.0) [25] on R-software (R version 4.3.0) [26]. Exact ASVs were inferred by DADA2 algorithm, and chimaeras were removed with default parameters. Taxonomy was assigned to ASVs up to the genus level with the SILVA database species train set file (version 138.1) [27]. Phyloseq R package (version 1.44.0) [28] was used to reorganise and manipulate the microbiota data. Microbiota richness and diversity were estimated by calculating Shannon and Chao1 indexes for each sample using the microbiome R package (version 1.22.0).

#### 2.3.2. Short-Chain Fatty Acids (SCFA)

SCFAs from faecal samples were analysed by gas chromatography (GC-FID) according to the protocol published by [29], with some modifications. The faeces were diluted with sterile water in a 1:5 proportion and vortexed for 1 min. Then, 2 mL was mixed with 5 mL of sulphuric acid (9.2 M), and a small amount of sodium chloride was added to remove any traces of water in the sample. Then, 0.4 mL of internal standard solution (52.9 mM 2-Methylhexanoic acid) and 2 mL of diethyl ether were added and vortexed for 1 min. Samples were centrifuged for 3 min at 3000× *g*-force, and the supernatant was added to the chromatography vials and injected into the Agilent GC7890B-5977B GC-FID with a multipurpose sampler with a SUPELCOWAX™ 10 Capillary GC Column (30 m × 0.25 mm × 0.25 μm). The oven temperature program was 90 °C for 1 min, ramped to 190 °C at a rate of 5 °C/min, and finally held to 250 °C for 30 min. Helium was used as carrier gas at a flow rate of 1 mL/min with an inlet temperature of 250 °C, and the injection volume was 2 μL. For the quantification of the volatile short-chain fatty acids, acetic acid (AA), propionic acid (PA), butyric acid (BA), valeric acid (VA), isovaleric acid (IVA), and isobutyric acid (IBA), analytical standards were used. Calibration lines were prepared ranging from 0 to 30 mM, and results were expressed (mmol/g).

### 2.4. Statistical Analyses

Different datasets were summarised by mean and standard deviation; first quartile, median, and third quartile for numerical variables; and absolute frequencies for categorical variables.

To compare the faecal microbiota results of the subjects on CF modulator therapy with the rest of the subjects, PERMANOVA analyses were performed based on the relative abundance of bacterial genera, setting 999 permutation distances calculated as Bray distances. The definitive *p*-value was estimated by calculating the mean of the resulting *p*-value from 100 tests. In addition, *t*-tests were made to assess significant differences between single variables, such as specific genera and alpha-diversity indices, and *p*-value adjustment by false discovery rate (FDR) was applied when necessary.

Correlations between continuous variables from different datasets were elucidated by multiple elastic net penalised regression. This consists of fitting a linear regression model within a restriction, i.e., a limitation of the value of parameters of explanatory variables. It causes the constriction of the coefficients towards zero, potentially annulling some variables in the model and selecting others [30]. Penalty factor lambda was selected as the most repeated value in 500 replicates of a 10-fold cross-validation. In every replicate, the selected lambda was one standard error from the minimum one (one standard error principle). Thus, selected variables were those with a penalised coefficient different from zero. Elastic net penalised regression was performed with glmnet R package (version 4.1.7) [31].

## 3. Results

### 3.1. Clinical Characteristics of the Subjects

The study cohort consisted of 43 children with CF with a mean age of 10.8 (4.9) years old, with a 24/19 proportion of male and female subjects (Table 2). Most of the participants (21/43) had Class II CFTR mutations in both alleles, of which 19/43 presented F508del in homozygosis. The anthropometric nutritional status indicators were defined by mean z-scores for weight, height, and body mass index (BMI) as follows: −0.2 (0.9), −0.4 (1.6), and −0.1 (1.1), respectively. The pulmonary function was characterised by a mean FEV1 of 89.8% (20.0). All the subjects were pancreatic insufficient, with a mean daily PERT dose of 10145.6 (18827.6) lipase units (LU/kg). Treatment with CFTR modulator therapy (Ivacaftor) had started in five of the subjects, who presented the F508del mutation in homozygosis.

### 3.2. Dietary Assessment

The median of food intake described in terms of food groups, macronutrients, and type of dietary fibre is presented in Table 3.

Moving on to food groups, milk and dairy represented the highest group in the daily intake (212.5 g/day), followed by refined cereals (174.4), meat (83.3). fruit (67.5), sugar-added dairy (60.0), and snacks and sweets (60.0). Inversely, whole-grain cereals, nuts and eggs were the lowest consumed group.

The diet in this cohort was characterised by a macronutrient proportion of 37.5–43.9–14.0% of the total daily energy intake from carbohydrates, lipids, and protein, respectively. Simple carbohydrates represented 14.6%, while saturated fatty acids were 15.1%. Focusing on dietary fibre, a median amount of 10.6 g/day was registered; from it, 3.8 g/day corresponded to hemicellulose, 1.2 g/day to pectin, 2.7 g/day to cellulose, and 1.2 g/day to lignin. Considering the specific fibre intake guidelines for this age, the percentage of compliance with this recommendation was variable.

**Figure 1:**
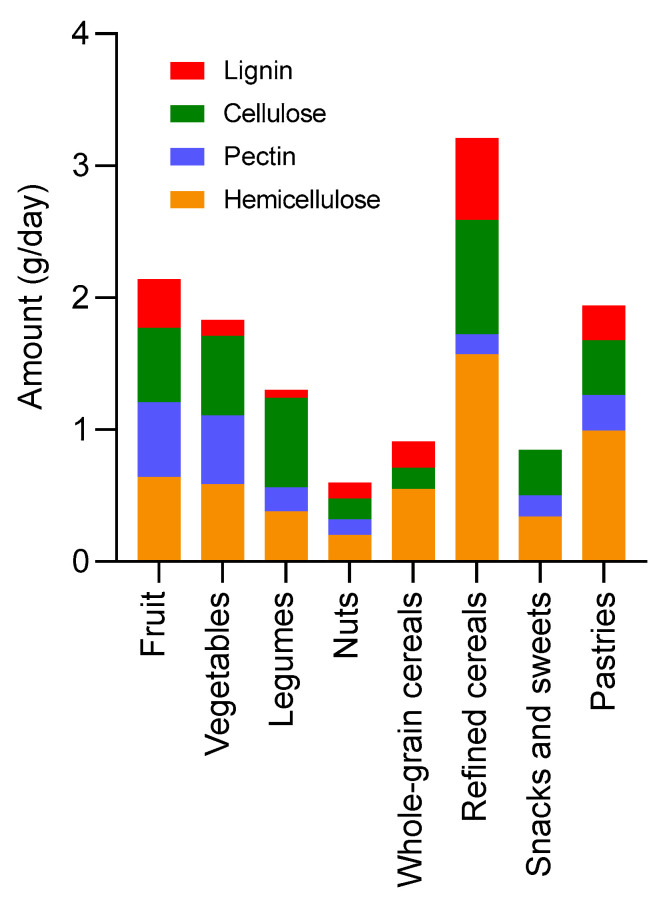
Contribution of food groups to the total fibre intake, including the fractions of the specific types of fibre.

**Figure 2:**
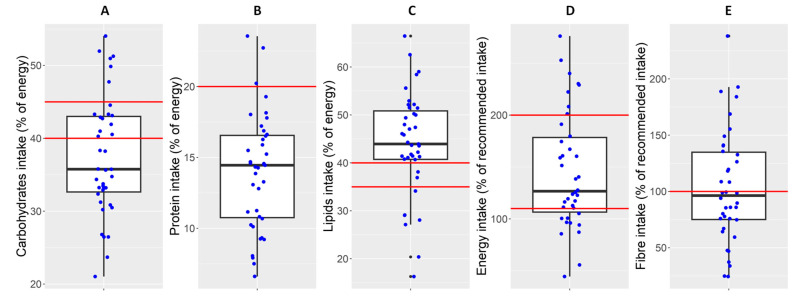
Compliance with the recommended intake of carbohydrates (**A**), protein (**B**), lipids (**C**), and energy (**D**), according to the ESPEN, ESPGHAN, and ECFS clinical guidelines for nutrition in CF [32], and fibre (**E**) referred to the FAO guidelines [33] (red lines), expressed with boxplots representing the median value (horizontal black lines) for each variable.

In addition to the descriptive overview, the contribution of the food groups to the intake of the different fibre types is presented in Figure 1. As observed, refined cereals were the group contributing the most to fibre intake, representing 3.2 g/day, followed by the group of pastries, with 1.9 g/day. Fruit, vegetables, and legumes accounted for 2.2, 1.8, and 1.3 g/day, respectively. Due to their low consumption, the groups showing the lowest contribution to fibre intake were nuts (0.6 g/day) and whole-grain cereals (0.9 g/day). In most of the food groups, pectin represented the lowest proportion, followed by lignin, while hemicellulose and cellulose were the majoritarian fibre types in the diet. Moving on to fibre intake by age, it was found to be as follows: 1–3 years, 4.7 g fibre/day; 4–6 years, 7.7 g fibre/day; 7–10 years, 9.9 g fibre/day; 11–14 years, 13.2 g fibre/day; 15–17 years, 22.3 g fibre/day; and ≥18 years, 8.3 g fibre/day. Only the group of 15–17-year-olds met the recommended intake (Table S2). Overall, compliance with the recommendations for macronutrients, energy, and fibre is represented in Figure 2.

### 3.3. Faecal Microbiota

**Figure 3:**
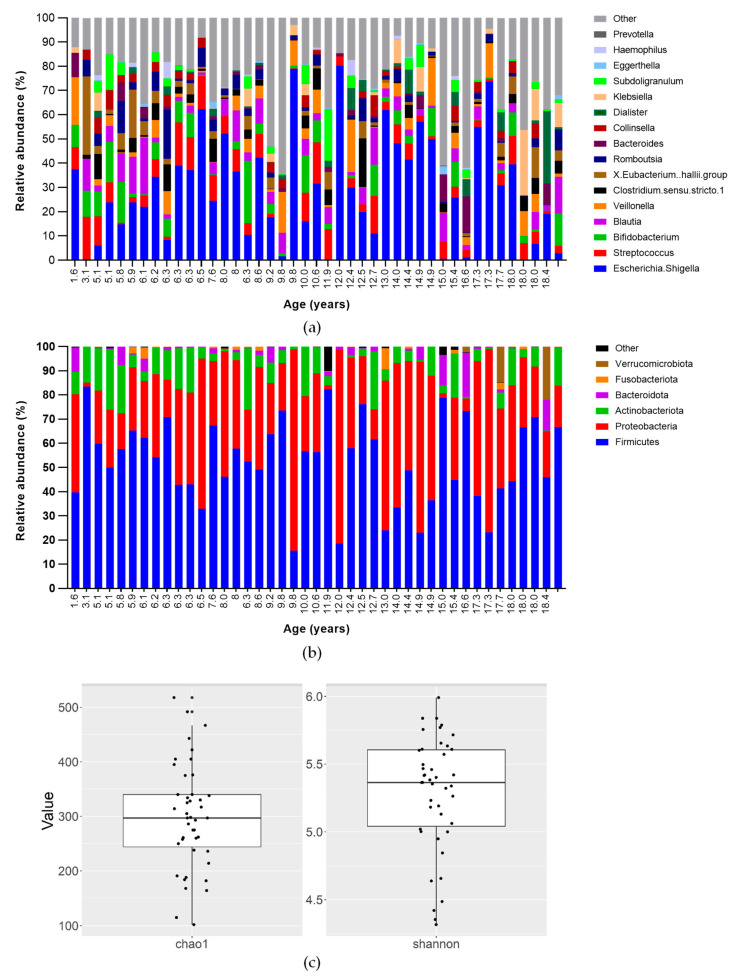
Relative abundance of faecal microbiota in the study cohort ordered by age at phylum (**a**) and genus level (**b**). Shannon index and Chao-1 express the alpha diversity of the faecal microbiota (**c**).

The faecal microbiota was characterised by the majoritarian presence of the phylum Firmicutes, which represented 54.5% of the relative abundance. The sample was further characterised by a mean abundance of 32.0% of Proteobacteria, 9.6% of Actinobacteriota, 2.2% of Bacteroidota, and 0.5% of Verrucomicrobiota (Figure 3a). At the genus level (Figure 3b), *Escherichia-Shigella* was the most abundant (27.4%). Other genera in relatively high proportions were *Streptococcus* (7%), *Blautia* (6.1%), *Bifidobacterium* (6.1%), *Veillonella* (4.9%), *Eubacterium hallii* (4.0%), *Clostridium sensu stricto* (3.5%), and *Subdoligranulum* (2.5%). At the other end of the spectrum, *Roseburia* (0.3%), *Lactobacillus* (0.7%), and *Faecalibacterium* (1.2%) were found. When expressing the microbiota results as alpha diversity, the Shannon index and Chao-1 were found to be 5.3 (0.4) and 299.1 (95.8), respectively (Figure 3c).

When considering the individuals on CF modulator therapy separately (n = 5), no significant differences (*p* = 0.383) with the rest of the study group were detected in terms of diversity (Shannon index). However, some bacterial genera presented with significant differences (adjusted *p*-values) in relative abundance in this subset of patients. In particular, *Bifidobacterium* was lower in patients on CF modulators (mean 0.72% vs. 6.31%, *p* < 0.0001), along with Streptococcus (mean 1.75% vs. 7.53%), *Clostridium sensu stricto 1* (0.43% vs. 3.75%), *Eubacterium hallii* group (0.27% vs. 4.06%), and *Anaerostipes* (0.26% vs. 1.56%) (*p* < 0.001) (Figure S1). In terms of phyla, the general profile at this taxonomic level did not show significant differences between children with CFTR modulator therapy and those without, except for Actinobacteriota, which was significantly lower in the modulator group (2.47% vs. 9.40%, *p* = 0.002).

### 3.4. Short-Chain Fatty Acids (SCFA)

The metabolic activity of faecal microbiota was expressed as the concentration of SCFA. According to Table 4, total SCFA was at a concentration of 25.1 mM, of which the linear species represented 89.8% and the branched-chain fatty acids were found at 10.2%. The SCFA that was found in the highest proportion was BA (10.4 mM), followed by AA (5.1), PA (3.5), and VA (0.2). Regarding the branched-chain series, IBA was found at 0.5 mM and IVA at 0.9 mM.

### 3.5. Correlations between Dietary Components and Faecal Microbiota

**Figure 4:**
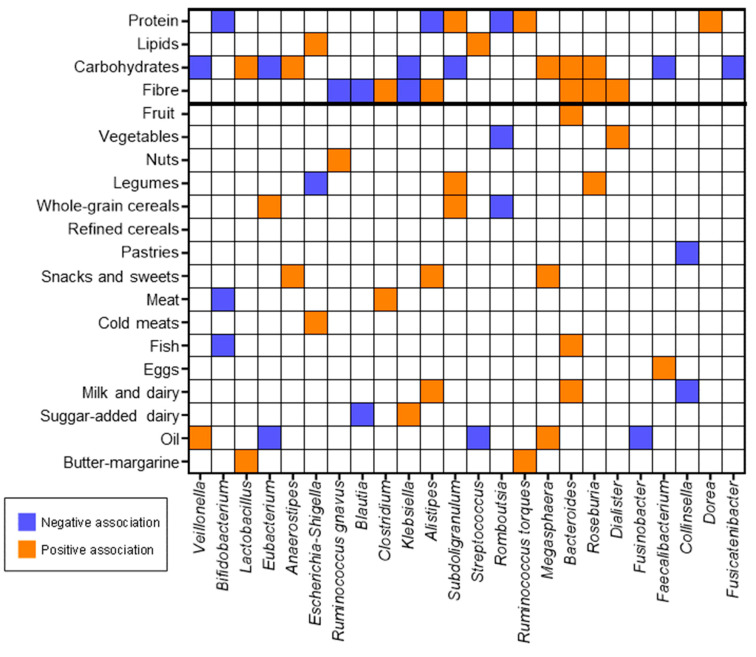
Significant associations between food groups, macronutrients, and fibre and faecal microbiota composition. The bold line separates the series of nutrients from the series of food groups.

Significant associations between food groups and faecal microbiota were found (Figure 4). Fruit, nuts, and vegetables presented a positive association with *Bacteroides*, *Rumminococcus* (gravus group), and *Dialister*, respectively. Legumes had a positive association with *Subdoligranulum* and a negative association with *Escherichia/Shigella.* Whole-grain cereals also presented a positive association with *Subdoligranulum* and *Eubacterium* and a negative association with *Romboutsia*. Snacks and sweets showed a positive association with *Anaerostipes*, *Alistipes*, and *Bacteroides*. Meat, cold meats, and fish showed positive associations with *Clostridium*, *Escherichia/Shigella*, and *Bacteroides*, respectively, and both meat and fish present negative associations with *Bifidobacterium*. Eggs were the only food group that showed a positive association with the genus *Faecalibacterium*. In the case of the milk and dairy group, it was positively associated with *Alistipes* and *Bacteroides*, while sugar-added dairy was associated only with *Klebsiella*. Milk and dairy and sugar-added dairy products presented negative associations with *Klebsiella* and *Blautia.* Oil presented a positive association with *Veillonela* and *Megasphaera* and a negative association with *Eubacterium* and *Streptococcus*. Finally, butter and margarine only presented a positive association with *Lactobacillus* and *Ruminococcus* (torques group).

The correlation analysis between macronutrients and microbiota showed that higher protein consumption was negatively associated with the abundance of *Bifidobacterium*, *Alistipes*, and *Romboutsia*. Inversely, this macronutrient showed a positive association with *Ruminococcus* (torques group), *Subdoligranulum*, and *Dorea*. The intake of lipids was found to have a negative association with *Escherichia/Shigella* and a positive association with *Streptococcus.* Carbohydrates showed most of the positive correlations, concretely with *Bacteroides*, *Lactobacillus*, *Megasphaera*, *Anaerostipes*, and *Roseburia.* In contrast, carbohydrates were negatively associated with *Veillonella*, *Eubacterium*, *Subdoligranolum*, *Faecalibacterium*, *Fusicatenibacter*, and *Klebsiella*. Fibre showed a positive association with *Bacteroides*, *Roseburia*, *Clostridium*, *Alistipes*, and *Dialister*, while *Ruminococcus* (torques group), *Blautia*, and *Klebsiella* were negatively associated with dietary fibre.

## 4. Discussion

This study has addressed for the first time the impact of diet on colonic microbiota in children with CF and its food-group origin. The results have enabled the identification of those dietary components and food groups associated with faecal-beneficial and pathogenic bacterial genera.

First, the analysis of the diet has confirmed that our cohort adhered to the “high-fat and high-energy” diet, as evidenced by the energy intake within 110–200% as compared to the healthy population and the total lipid intake being higher than the currently recommended 35–40% for nutrition in CF [32]. Additionally, the results confirm the findings of previous studies [21], that energy and fat intake were achieved at the expense of an unhealthy lipid profile (>10% of SFA) [33,34], a high intake of simple carbohydrates, and a high representation of food groups like snacks and sweets or refined cereals [35].

In terms of dietary fibre, the recommended intake by age was not met in most of the cases. This result is supported by the fact that relevant fibre sources, such as nuts or whole-grain cereals, contributed only in minoritarian proportions, and fruit and vegetables were not the major sources either. Indeed, fibre intake was achieved mainly from the intake of refined cereals, a group characterised by the low content of dietary fibre. This result is evidence that low-fibre foods are overconsumed. Altogether, the study findings suggest that the “high-fat, high-energy” diet is unsuitable for promoting healthy sources of dietary fibre, which could contribute to dysbiosis in colonic microbiota [36,37].

Focusing on colonic microbiota, the results were in accordance with previous studies conducted in children with CF, including a low alpha diversity index compared to healthy children [38], unbalanced proportions between Bacteroidota and Proteobacteria, and the presence of bacterial genera. On the other hand, the production of SCFA in our cohort was low (i.e., 25 mM), compared to previous studies with healthy children [39,40].

Regarding the effect of CFTR modulators on gut microbiota, one study reported that, after treatment with Ivacaftor, there were no significant changes in the composition but diversity increased. Another study showed that Ivacaftor was able to induce an increase in the relative abundance of *Akkermansia*, while there were no significant changes in diversity, suggesting that alterations in the composition of the microbiota could not only be affected by factors related to CTFR but also by others, such as diet [17,41]. In our study, it was evident that there were significant differences between the composition of *Bifidobacterium*, *Streptococcus*, *Clostridium sensu stricto 1*, *Eubacterium hallii* group, and *Anaerostipes* in children who received treatment with modulators versus those who did not receive treatment. However, the sample size (n = 5) of children with CFTR modulators is considered a limitation, so this result should be interpreted with caution.

Significant associations were found when assessing how diet could contribute to colonic microbiota composition and metabolic activity. These associations should be interpreted with caution, as not only diet but also other known factors contribute to gut dysbiosis, such as the use of antibiotics, the lung–gut axis, and the altered intestinal conditions occurring in CF [42,43]. Furthermore, it must be considered that our study design is observational, which limits the possibility of attributing the effect of a specific dietary component to the composition of colonic microbiota.

In this sense, the negative association between protein intake and *Bifidobacterium* and *Romboutsia* coincides with previous studies [44,45,46,47], which could be related to the inability to utilise the unabsorbed protein as substrates against other genera that could more efficiently shift from carbohydrate to protein degradation [48]. Additionally, both meat and fish intake, some of the main protein sources, were negatively associated with *Bifidobacterium*. Other studies evidenced that lipids were significantly associated with reduced *Faecalibacterium* and *Akkermansia* [49,50], which could not be found in our study, possibly because both genera were in negligible proportions. Within carbohydrates, some structures, such as resistant starch, are known to be the most fermentable by colonic microbiota [51]. Its presence promotes an increase in beneficial microbial groups and a decrease in pathogenic bacteria [52], as shown in our cohort (e.g., positive associations with *Bacteroides* or *Lactobacillus* and negative associations with *Veillonella* or *Klebsiella*). Regarding total dietary fibre, the positive association between *Bacteroides* and *Roseburia*, both known for their ability to ferment indigestible carbohydrates, is coherent with the current evidence [53].

In particular, maldigestion and malabsorption of nutrients, including lipids and proteins, could be mainly behind the altered interaction of microbiota with dietary components. Malabsorption differs from the regular digestion process, in which macronutrients are digested and absorbed in the upper gastrointestinal tract, so only minoritarian proportions reach the colon. In contrast, dietary fibre reaches the colon with an unaltered structure, being available as substrates for colonic microbiota [54]. In CF, the unabsorbed macronutrients reach the colon in much larger proportions [55].

Despite there being no specific studies on the effect of malabsorption on colonic microbiota, an analogous situation would be the context of “high fat diets”, in which fat intake exceeds the rate of digestion and absorption in the small intestine and enters the colon [56], as in the case of malabsorption in CF. Indeed, a previous study showed that switching to a high-fat diet leads to increased Proteobacteria and decreased Bacteroidota [57], coinciding with the findings of our study. The mechanisms by which “high-fat diets” promote changes in colonic microbiota are not well-defined yet [58]. Possible explanations include that the growth of Proteobacteria could be related to the ability of this phylum to use as substrate the glycerol polar head of triglycerides [59]. Thus, considering this rationale, in the context of CF, not only are lipids overconsumed due to the “high-fat, high-energy diet” recommendations but also, these lipids (and proteins) are less efficiently digested and absorbed [60], which could lead to additional alterations in colonic microbiota. The positive association between lipid intake and *Escherichia/Shigella* (Proteobacteria) supports this explanation. From another point of view, excess fat in the small intestine could have interacted with dietary fibre [61].

As for the limitations, the study was conducted with the methodology of 16S sequencing to assess faecal microbiota. The alternative would have been shotgun metagenomics, which presents some advantages against 16S, such as inferring functional information due to specific gene detection and quantification or identifying bacterial taxa with a greater resolution, even at the species level. Nevertheless, we chose 16S sequencing because it is well suited for the analysis of a large number of samples, i.e., multiple patients, although it offers limited taxonomical and functional resolution [62].

Altogether, the study findings are relevant due to the particular diet and the specific digestion and absorption alterations in children with CF. Based on the results, the importance of switching from refined cereals or snacks and sweets as a source of dietary fibre to other food groups, such as legumes, whole-grain cereals, nuts, fruit, and vegetables, is highlighted. In this sense, the message of this study encourages the set-up of further clinical trials with an interventional design to assess the role of moving from the “high-fat high-energy” diet to the “standard healthy diet” or the “high-fibre diet” on modifying colonic microbiota and SCFA production in children with CF.

In conclusion, the unbalanced diet of children with CF, including excessive fat and simple carbohydrate intake and inadequate sources of dietary fibre, would contribute to the unbalanced faecal microbiota profile. The identified association between protein and reduced *Bifidobacterium*, lipids and increased *Escherichia*/*Shigella*, and carbohydrates and reduced *Veillonella* and *Klebsiella* deserves confirmation in future interventional studies.

## Figures and Tables

**Table 1:** Food group classification and examples.

Name of the Group	Examples
Milk and dairy	Yoghurt, milk, cheese, etc.
Sugar-added dairy	Sweetened yoghurt, milkshakes, milk-based desserts, etc.
Fruit	All fresh fruit, dried fruit
Vegetables	All fresh vegetables
Legumes	All legumes
Nuts	All nuts
Whole-grain cereals	Bread, rice, pasta, etc.
Refined cereals	Bread, rice, pasta, boiled potato, etc.
Snacks and sweets	Chocolate, candies, chips, cocoa powder, sweeteners, others, for example, ultraprocessed foods
Meat	Fresh beef, chicken, pork, rabbit, etc.
Cold meats	Sausages, hamburgers, nuggets, ham, etc.
Fish	Seafood, white fish, blue fish, canned fish, etc.
Eggs	All eggs
Oils	Olive oil
Butter/margarine	Butter and margarine
Pastries	Regular cookies, chocolate cookies, breakfast cereals, doughnuts, cupcakes, etc.

**Table 2:** Clinical and demographic data of the study cohort (n = 43).

Age (years) (mean (SD))	10.8 (4.9)
Sex (m/f)	24/19
Height (z-score) (mean (SD))	−0.4 (1.6)
Weight (z-score) (mean (SD))	−0.2 (0.9)
BMI (z-score) (mean (SD))	−0.1 (1.1)
FEV1 (%)(mean (SD))	89.8 (20.0)
Pancreatic insufficiency (n)	43
PERT dose (LU/day*kg) (mean (SD))	10,145.6 (18,827.6)
CFTR modulator therapy (n)	5

**Table 3:** Daily intake expressed as energy, food groups, macronutrients, and dietary fibre intake (Q1, median, Q3).

		Q1	Median	Q3
Energy (%)	Daily intake	106.6	126.7	178.5
Food groups (g/day)	Milk and dairy	119.3	212.5	439.5
	Sugar-added dairy	0.0	60.0	182.0
	Fruit	0.5	67.5	131.7
	Vegetables	28.3	52.5	120.3
	Legumes	0.0	10.0	49.3
	Nuts	0.0	0.0	0.0
	Whole-grain cereals	0.0	0.0	0.0
	Refined cereals	115.9	174.4	237.1
	Snacks and sweets	20.3	60.0	139.2
	Meat	44.0	83.3	135.0
	Cold meats	20.5	40.0	74.4
	Fish	0.0	26.7	50.0
	Eggs	0.0	0.0	13.9
	Oil	1.4	4.7	17.5
	Butter/Margarine	0.0	0.0	0.0
	Pastries	15.2	48.8	63.8
Macronutrients (% from daily energy intake)	Carbohydrates	32.6	35.8	43.0
	Simple carbohydrates	9.6	14.1	18.4
	Complex carbohydrates	15.5	19.6	22.8
	Lipids	40.7	43.9	50.9
	SFA	12.2	13.9	16.9
	MUFA	15.7	17.8	23.8
	PUFA	4.9	7.1	8.8
	Protein	10.8	14.5	16.6
Dietary fibre (g/day)	Total dietary fibre	5.3	9.1	13.8
	Total insoluble fibre	4.2	7.4	11.2
	Insoluble cellulose	1.4	2.2	3.4
	Insoluble hemicellulose	1.3	1.8	3.5
	Insoluble pectin	0.3	0.6	0.9
	Insoluble lignin	0.6	0.9	1.5
	Total soluble fibre	1.2	1.8	2.7
	Soluble hemicellulose	0.7	1.1	1.7
	Soluble pectin	0.2	0.4	0.5

**Table 4:** Quantification of short-chain fatty acids (SCFA) in faecal samples.

Metabolites	Concentration (mM)		
	Q1	Median	Q3
Total SCFA	19.6	25.1	34.2
Acetic acid (AA)	4.1	5.1	9.9
Butyric acid (BA)	6.5	10.4	12.5
Propionic acid (PA)	2.4	3.5	4.9
Valeric acid (VA)	0.1	0.2	0.7
Total linear SCFA	16.5	19.9	33.1
Isobutyric acid (IBA)	0.3	0.5	1.5
Isovaleric acid (IVA)	0.6	0.9	2.7
Total branched SCFA	0.9	1.5	4.4

## Data Availability

Data will be made available on request.

## Supplementary Materials

The following supporting information can be downloaded at: https://www.mdpi.com/article/10.3390/nu15245013/s1, Table S1: Database of different types of dietary fibre (hemicellulose, pectin, cellulose, and lignin) of typical consumed foods; Table S2: Daily intake of fibre of children with cystic fibrosis vs. recommended fibre intake by age; Figure S1: Relative abundance of microbiota of children who received treatment with modulators versus those who did not receive treatment at genera level with statistically significant differences.
